# Microstructural Deformation and Failure of Highly Explosive-Filled Polymer Composites Under Dynamic Compression

**DOI:** 10.3390/polym17070867

**Published:** 2025-03-24

**Authors:** Xiaowei Zhang, Heming Zhao, Wanqian Yu, Qiao Zhang, Yi Sun, Youcai Xiao

**Affiliations:** 1 College of Mechatronic Engineering, North University of China, Taiyuan 030051, China; hgdzhangxiaowei@163.com (X.Z.); 19834049591@139.com (Q.Z.); 2 National Key Laboratory of Land & Air Based Information Perception and Control, Xi’an Modern Control Technology Research Institute, Xi’an 710065, China; hsywq812907@163.com; 3 Departments of Astronautic Science and Mechanics, Harbin Institute of Technology, Harbin 150001, China

**Keywords:** highly particle-filled polymer bonded explosive, cohesive zone model, micromechanical behavior, interface properties, damage characteristics

## Abstract

The dynamic mechanical properties and damage behaviors of polymer-bonded explosives (PBXs), as a kind of highly particle-filled polymer composite, must be known to ensure the safe use of related weapons and munitions. The high particle volume fraction of PBXs, which can reach approximately 95%, makes it difficult to investigate their mechanical properties and damage behavior via conventional methods. In this study, a microstructural model was developed by employing the Voronoi correction method to achieve a highly particle-filled PBX. Additionally, a bilinear model was used to accurately represent the nonlinearity of the stress–strain curve, while a zero-thickness cohesive zone model was incorporated to effectively describe the damage mechanism. The dynamic mechanical properties and damage behavior of PBXs with high particle fractions were elucidated to comprehensively understand the effects of strain rate, interface strength, and particle volume fraction on peak stress, failure strain, and damage extent. The numerical results exhibit excellent concurrence with existing experimental measurements and other computational simulations. The mechanical behavior of PBXs was also described by developing a viscoelastic model based on damage, which incorporated the equations associated with macroscopic and microscopic damage evolution. Overall, the proposed numerical technique is effective for comprehending the mechanical behavior and microscopic damage response of PBXs subjected to dynamic compression.

## 1. Introduction

Polymer-bonded explosives (PBXs) are highly explosive-filled polymer composites that generally comprise high-energy single-compound particles (e.g., RDX and HMX), polymer binders (e.g., HTPB and Estane), and particle fractions of 60% to 95% by weight [1,2]. PBXs have found extensive application in both the military and civilian sectors, including the manufacturing of missiles and rockets [3,4]. When PBXs are subjected to impact, vibration, and shock, they may undergo damage and their mechanical properties can deteriorate, thus increasing the ignition sensitivity and hampering their safety and reliability [5,6,7]. Thus, a comprehensive understanding of the mechanical characteristics, detrimental effects, and progression patterns of damage in PBXs is necessary [8,9]. This study presents a comprehensive investigation into the mechanical behavior of PBXs under dynamic compression. While exploring their extensive potential, it is essential to address the dual-use concerns and potential threats associated with PBXs. Specifically, although the application of high explosives offers significant improvements in operational efficiency, system performance, and safety standards within the defense sector, the inherent risk of their diversion for illicit purposes may lead to detrimental societal impacts and pose substantial safety challenges.

However, this research holds significant scientific merit by advancing our fundamental understanding of the mechanical properties of PBXs and driving technological innovation in the field. These developments not only broaden our fundamental understanding of energetic material dynamics but also facilitate the engineering of advanced explosive formulations with optimized performance characteristics. Moreover, a comprehensive understanding of the mechanical behavior of PBXs is crucial for implementing sophisticated safety protocols and developing effective risk management strategies. We have concluded that the potential benefits and positive implications of this study significantly outweigh the disadvantages. We hereby solemnly affirm that this research has undergone strict institutional review and has obtained official approval from the Ethics Committee of North University of China. The institutional review board carried out a comprehensive assessment of the research methodology, experimental protocols, and potential societal impacts, verifying full compliance with international academic integrity standards and institutional ethical guidelines. As principal investigators, we assert our commitment to implementing continuous safety monitoring protocols and maintaining rigorous control measures throughout all practical applications of this material. Furthermore, we pledge to promote scientific knowledge through accountable research practices while contributing to sustainable technological innovations that are in accordance with societal welfare and global security considerations.

In recent years, researchers have studied the mechanical properties and failure mechanisms of PBXs via quasi-static and dynamic experiments. The microstructural deformation and damage mechanisms of PBXs under quasi-static tension have been investigated by Brazilian experiments and real-time microscopic observations, which have revealed that interfacial debonding and crystalline cracks are present in the observed failure modes [5,10,11]. Skidmore et al. [12] and Chen et al. [6] employed uniaxial compression experiments to investigate the microstructural evolution and damage mechanism of PBXs under quasi-compression conditions, thus revealing the significant involvement of crystal fracture during the damage process. Rae et al. [13] and Goldrein et al. [14] used a Moore interferometer to measure the deformation and strain fields within the central micro-region of a disc model, thereby providing further insights into the deformation and damage processes of PBXs. By using a digital image correlation (DIC) technique, Zhou et al. [15] demonstrated that the failure of PBXs during the three-point bending experiment of a half-disk model was primarily attributed to interfacial debonding. Meanwhile, the damage evolution mechanism of PBXs under uniaxial compression was elucidated by Liu et al. [16] using DIC as well. Numerous studies have demonstrated that debonding damage is prevalent in PBXs at low strain rates, whereas trans-crystalline fracture is the primary damage mode at high strain rates. Wu et al. [17] showed that an increase in strain rate facilitated the fracture of crystal particles. Zhang et al. [18] indicated that with increasing strain rates, interfacial debonding eventually led to the coexistence of multiple damage patterns. Chen et al. [19] and Ravindran et al. [20] investigated the mechanical properties and damage mechanisms of different substituted materials via dynamic loading experiments.

Although this experimental method can elucidate the relationship between the microstructures and macroscopic mechanical responses of materials from a phenomenological perspective, it poses challenges in revealing material failure mechanisms at a microscopic level. With the advancement of numerical techniques, researchers have utilized the finite element method (FEM) [21], the extended finite element method (XFEM) [22], the discrete element method (DEM) [23], the numerical manifold method (NMM) [24], the cohesive zone model (CZM) [25,26], phase-field models (PFMs) [27], and peridynamics (PD) [28] to study particle-filled composites. Zhang et al. [21] discussed the effects of particle shape, particle clustering, and debonding on the crack extension and fracture toughness of a material using a finite element model. Kang et al. [24] investigated the microstructures and effective moduli of PBXs using a numerical manifold method. Xiao et al. [26] investigated the mechanical properties and damage evolution behavior of PBXs with low particle content under a high strain rate by using a CZM. Wang et al. [29] developed a damage elastic–plastic model to study the deformation and damage mechanisms of PBXs under dynamic loading. Existing research on the damage and failure mechanisms of PBXs has emphasized microscale calculations under quasi-static conditions, while studies performed under dynamic conditions have been relatively scarce due to the lack of suitable constitutive models that can be used for characterizing the dynamic damage effects of energetic crystals, binders, and interfaces.

In this study, the CZM was employed to investigate the compression damage behaviors of PBXs with high particle fractions under high strain rates. The mechanical responses and failure mechanisms of PBXs under external loading were analyzed. Furthermore, the effects of strain rate, interfacial strength, and particle volume fraction on the mechanical properties and damage evolution behavior of PBXs were examined while quantifying the changes in damage percentages and total crack length at different locations (particle, matrix, and particle–matrix) influenced by various factors. A viscoelastic model incorporating damage was developed to accurately describe the mechanical behavior of PBXs.

## 2. Constructive Model

### 2.1. Elastic Model for Particles

Previous studies [30,31] have shown that RDX particles undergo fracture when subjected to compression–shear loading while exhibiting minimal plastic deformation. To characterize the mechanical properties of these particles, an elastic model was utilized whose Young modulus and Poisson ratio were 18,400 MPa and 0.25, respectively.

### 2.2. Viscoelastic Model for Polymer Binder

The viscoelastic properties of polymer binders were effectively characterized by employing the generalized Maxwell model (GMM). Through the Boltzmann Superposition Principle (BSP) [32], the viscoelastic stress at moment t was calculated in the form of a viscoelastic strain integral, Equation (1) [33]:

(1) $$\sigma_{ij}(t)=\int_{0}^{t}C_{ijkl}(t-\tau )\frac{\partial \varepsilon_{kl}(\tau )}{\partial \tau }d\tau$$

where $\sigma_{ij}(t)$ is the instantaneous stress, *τ* is the time of applied deformation, and *C_ijkl_* is the relaxation modulus tensor. For each isotropic viscoelastic material, the fourth-order relaxation tensor, *C_ijkl_*, can be expressed using Equation (2):

(2) $$C_{ijkl}=\left(K(t)-\frac{2}{3}G(t)\right)\delta_{ij}\delta_{kl}+G(t)(\delta_{ik}\delta_{jl}+\delta_{il}\delta_{jk})$$

where *K*(*t*) and *G*(*t*) represent the bulk modulus and shear modulus, respectively. Based on GMM, *K*(*t*) and *G*(*t*) can be expressed in terms of Prony coefficients:

(3) $$\left\{\begin{array}{c} G(t)=G_{\infty }+\sum_{i=1}^{n}G_{i}\exp(-\frac{t}{\tau_{i}})\\ K(t)=K_{\infty }+\sum_{i=1}^{n}K_{i}\exp(-\frac{t}{\tau_{i}}) \end{array}\right.$$

where *K*_∞_ and *G*_∞_ are the long-term bulk and shear modulus, respectively. *K_i_* and *G_i_* are the *n*th shear and bulk relaxation modulus, respectively. *τ_i_* is the *n*th bulk and shear relaxation time, and *n* is the number of Maxwell elements. The 15th-order Prony series data (Table 1) were in agreement with the data in the existing literature [26,34]. *K_i_* and *G*_i_ can be obtained by *E*_i_. $K_{i}=\frac{E_{i}}{3(1-2\nu )}$ and $G_{i}=\frac{E_{i}}{2(1+\nu )}$, where the Poisson ratio (ν) is 0.45.

### 2.3. Bilinear Cohesive Law for Interface

**Figure 1:**
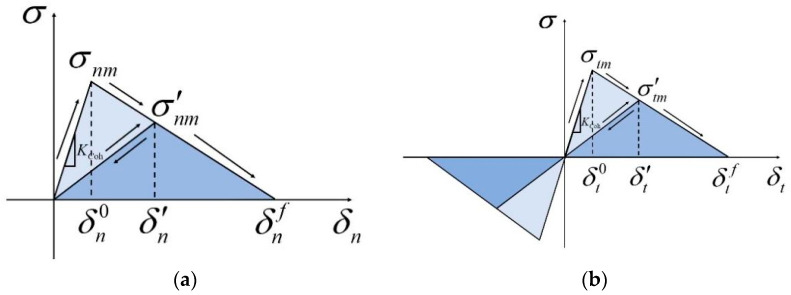
Bilinear traction–separation law model for cohesive elements: (**a**) mode I; (**b**) mode II and mode III.

In the CZM, cohesive elements are inserted between the surfaces of two neighboring grid cells of a finite element; however, the interface is governed by the relationship between the interfacial force and the degree of interfacial separation, and the following interfacial relationships are observed: bilinear, polynomial, and nonlinear [34,35,36]. For highly filled materials such as PBXs, a bilinear traction–separation law is generally used to describe the nonlinear relationship between traction and separation in the CZM [37,38]. Figure 1 shows a schematic of the bilinear cohesive law where $\delta_{n}^{0}$ and $\delta_{t}^{0}$ are the initial damage points and $\delta_{n}^{f}$ and $\delta_{t}^{f}$ are the final failure separation points of the model. The damage evolution mechanism is activated when stress reaches the critical values of $\sigma_{nm}$ and $\sigma_{tm}$:

(4) $$\max\left\{\frac{\langle \sigma_{n}\rangle }{\sigma_{nm}},\frac{\langle \sigma_{t}\rangle }{\sigma_{tm}}\right\}=1$$

where $\sigma_{n}$ and $\sigma_{t}$ represent the normal and shear stress of the cohesive element, respectively. Meanwhile, ⟨ ⟩ is the Macaulay bracket and defined as follows:

(5) $$\langle \sigma \rangle =\left\{\begin{array}{c} \sigma ,\sigma \geq 0\\ 0,\sigma <0 \end{array}\right.$$

The equations below govern the process of separating the cohesive element:

(6) $$\sigma_{n}=\left\{\begin{array}{c} \sigma_{nm}\frac{\delta }{\delta_{n}^{0}},0\leq \delta \leq \delta_{n}^{0}\\ \sigma_{nm}\frac{\delta_{n}^{f}-\delta }{\delta_{n}^{f}-\delta_{n}^{0}},\delta_{n}^{0}<\delta \leq \delta_{n}^{f} \end{array}\right.$$

(7) $$\sigma_{t}=\left\{\begin{array}{c} \sigma_{tm}\frac{\delta }{\delta_{t}^{0}},0\leq \delta \leq \delta_{t}^{0}\\ \sigma_{tm}\frac{\delta_{t}^{f}-\delta }{\delta_{t}^{f}-\delta_{t}^{0}},\delta_{t}^{0}<\delta \leq \delta_{t}^{f} \end{array}\right.$$

The cohesive element fails when the cracking displacements equal the failure displacements of $\delta_{n}^{f}$ and $\delta_{t}^{f}$.

In this study, we simulated a highly particle-filled PBX material based on several experimental and simulation studies that have calibrated and determined the interfacial parameters of the components (particles, binder, and particle–binder interface) [38,39], which include our previous work [26]. Table 2 shows all the parameters used.

## 3. Modeling the Micromechanics of PBXs

### 3.1. Numerical Simulation Model

**Figure 2:**
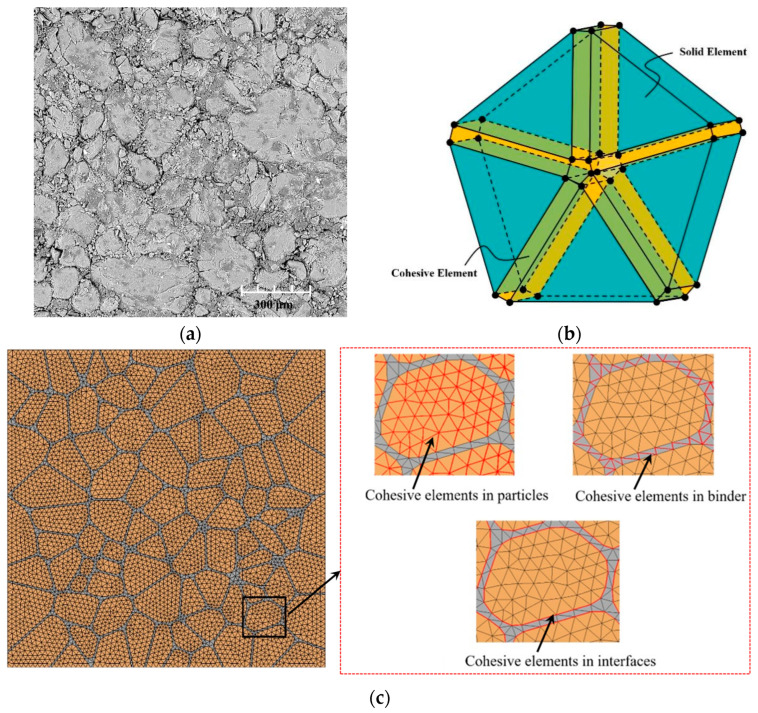
(**a**) The PBX microstructure (particle volume fraction = 87.39%); (**b**) cohesive elements that were reset and inserted; and (**c**) cohesive elements embedded in the particles, polymer matrix, and particle–matrix interface.

Scanning electron microscopy revealed that the PBX microstructure (Figure 2a) has several particles embedded within the polymer matrix. The samples utilized in this experiment were provided by the Xi’an Modern Chemistry Research Institute under China North Industries Group Co., Ltd., Xi’an, China. Each sample had a diameter of 6 mm and a thickness of 3 mm and was fabricated using a mold-pressing technique. Based on the PBX microstructure, we utilized the Voronoi correction method to generate a microstructure mode with a particle fraction of 87.39%, wherein the particles were randomly dispersed (Figure 2b). The models exhibited a length, width, and thickness of 2 mm, 2 mm, and 0.02 mm, respectively, while the comprising particles were sized 50 μm to 300 μm. The top of the microscopic model was subjected to a compressive displacement load, while the bottom was fixed and constrained in only the Z direction (with freedom in the X and Y directions) [26]. The microscopic structure contained three components: highly filled irregular particles, the polymer matrix, and the particle–matrix interface. Meanwhile, cohesive elements were embedded in the particles, polymer matrix, and particle–matrix interface, and the embedding process was explained using an example of a node shared by five triangular elements. The first step involved duplicating and resetting six individual nodes to the same location. Subsequently, these reset nodes were connected along the edges of the triangular cell to generate five cohesive elements of zero thickness. The three embedded condensate elements are highlighted in red (Figure 2c).

### 3.2. Mesh Size Study

**Figure 3:**
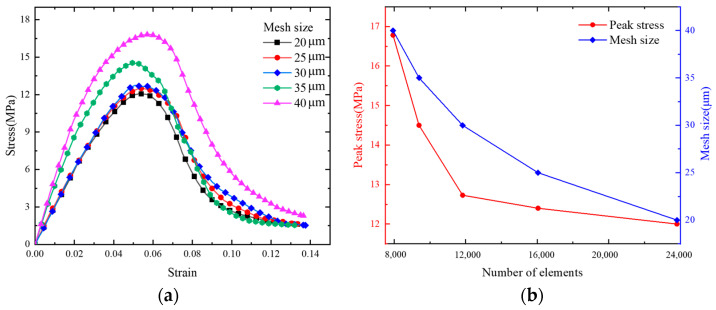
(**a**) Stress–strain curves for different mesh sizes at 1100 s^−1^; (**b**) number of meshes and peak stress for different mesh sizes.

The convergence of the numerical model was studied using five mesh sizes (20, 25, 30, 35, and 40 μm) to determine the optimal mesh size and number of meshes. Figure 3a shows the stress–strain curves corresponding to the different mesh sizes. Figure 3b shows the convergence of the number of mesh elements and peak stress for the different mesh sizes. The maximum stress typically reaches a convergence point when the mesh size is set to 30 μm and the total number of meshes becomes 11,827. By taking into account the balance between computational efficiency and precision, we opted for a mesh size of 30 μm in subsequent simulations.

### 3.3. Particle Size Study

**Figure 4:**
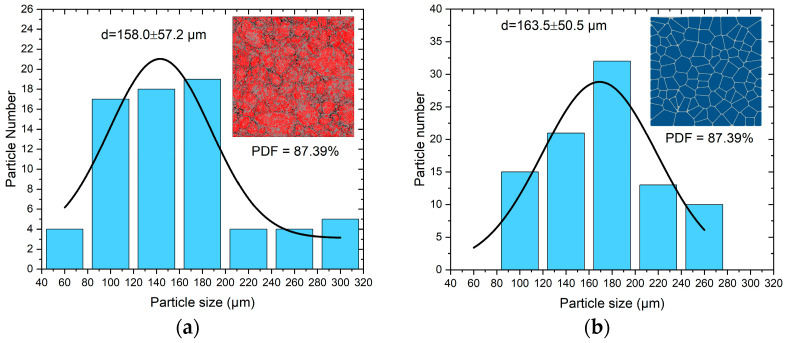
(**a**) The grain size distribution of the PBX microstructure with a grain volume fraction of 87.39% and (**b**) the grain size distribution of the model.

The effect of particle shape and size has been experimentally studied in the literature. Specifically, Heijden et al. [40] showed that grain content, size distribution, and smoothness of grains affect the initiation pressure of RDX- and HMX-based PBXs. As shown in Figure 4a, the mean grain size is 158.0 μm with a standard deviation of 57.2 μm in the PBX microstructure. The grain size distributions for the model are similar and have means of 163.5 μm and standard deviations of 50.5 μm, as shown in Figure 4b. The particles have multifaceted irregular shapes and are distributed randomly. Most PBX composites are essentially isotropic at scales above several interparticle distances. Note that in the generation of microstructures using Voronoi tessellation, energetic granules are effectively grown in place, subject to spatial constraint, whereas in actual PBXs, the grains are grown in solution and pressed or cast to the desired density and composition.

**Figure 5:**
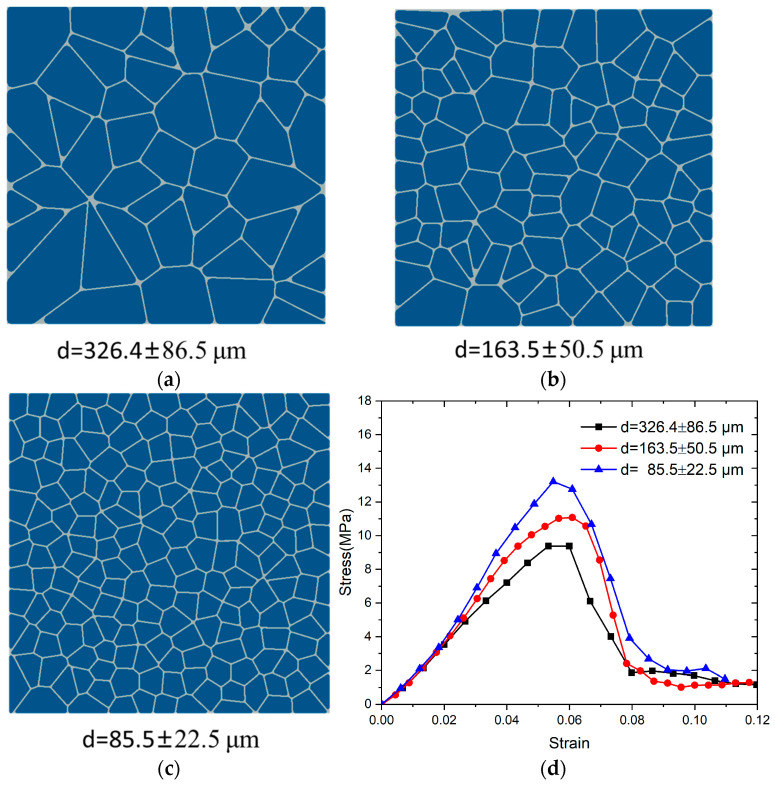
(**a**) Microscopic models with particle sizes of 326.4 ± 86.5 μm, (**b**) 163.5 ± 50.5 μm, and (**c**) 85.5 ± 22.5 μm and (**d**) the stress–strain curves for three different particle sizes at a strain rate of 1000 s^−1^.

Figure 5a–c illustrates microscopic models with particle sizes of 326.4 ± 86.5 μm, 163.5 ± 50.5 μm, and 85.5 ± 22.5 μm, respectively, and the corresponding particle counts are 200, 100, and 50. Figure 5d illustrates the stress–strain curves for three different particle sizes under compressive loading at a strain rate of 1000 s^−1^. The particle size of the PBX has a negligible influence on the effective elastic modulus of the PBX materials, in line with previous studies [41]. When the volume fraction of the particles remains constant, smaller particle sizes exhibit higher yield strengths in PBX composites. In the microscopic model consisting of 200 particles, the maximum principal compressive stress reached 13.9 MPa, representing a 20.9% increase relative to the model with 50 particles. Smaller size distributions of grains are beneficial to the load-carrying-capacity of PBXs under dynamic loading due primarily to enhanced grain–grain interactions and efficient packing of smaller grains between larger grains. This result indicates that PBXs exhibit a significant particle size effect [42].

### 3.4. Stress Equilibrium and Model Verification

**Figure 6:**
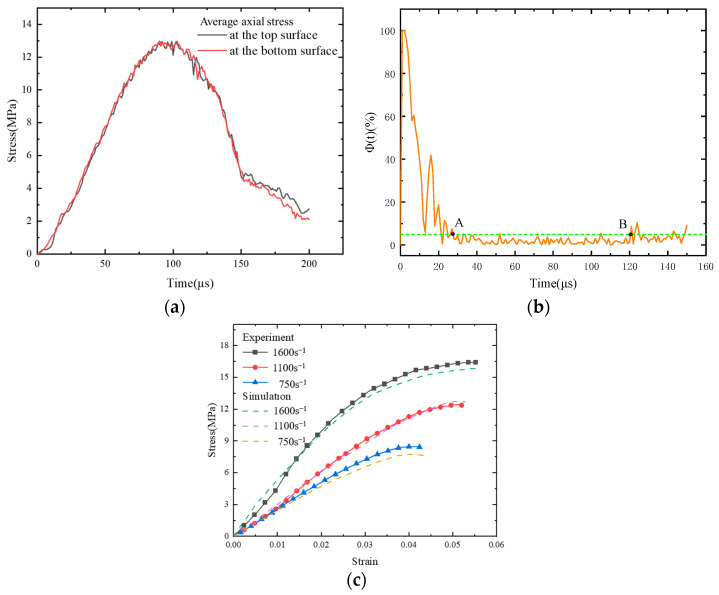
(**a**) Average axial stress history curves for the top and bottom surfaces; (**b**) difference between the relative stresses on the top and bottom surfaces; and (**c**) comparison of experimentally obtained and numerically simulated stress–strain curves.

The stress history curves depicted in Figure 6a for the upper and lower surfaces of the numerical model exhibit a striking resemblance to those reported in the literature [43]. The relative stress difference between the top and bottom surfaces was calculated using Equation (8):

(8) $$\Phi \left(t\right)=\left|\frac{\Delta \sigma (t)}{\sigma_{T}(t)}\right|$$

where Δσ(t) is the stress difference between the top and bottom surfaces and $\sigma_{T}(t)$ is the upper surface stress. It is assumed that the specimen will reach stress equilibrium when Φ(*t*) ≤ 5% [44]. The calculated relative stress difference is illustrated in Figure 6b, which shows that the value of the relative stress difference decreases as the loading time increases. This figure shows that the Φ(*t*) ≤ 5% condition is achieved between points A and B. Consequently, it can be concluded that the numerical model satisfies the stress equilibrium condition.

We performed numerical simulations for different loading strain rates by varying the loading rate. Figure 6c shows that the experimentally obtained stress–strain curves [43] are in good agreement with the numerically simulated stress–strain curves for the different loading strain rates. The relative errors between the simulation results of the model and the experimental data were less than 2% at strain rates of 1600 s^−1^ and 1100 s^−1^ and below 10% at a strain rate of 750 s^−1^. When impact velocity is low, the stress pulse generated may lack sufficient intensity or duration, leading to a reduced strain rate in the specimen. When the strain signal on the incident bar is exceedingly small, the associated test error tends to become relatively pronounced due to the limited signal-to-noise ratio. Therefore, our proposed model can be used for numerical simulations at high strain rates.

## 4. Results and Discussion

### 4.1. Mechanical Response and Failure Mechanism

**Figure 7:**
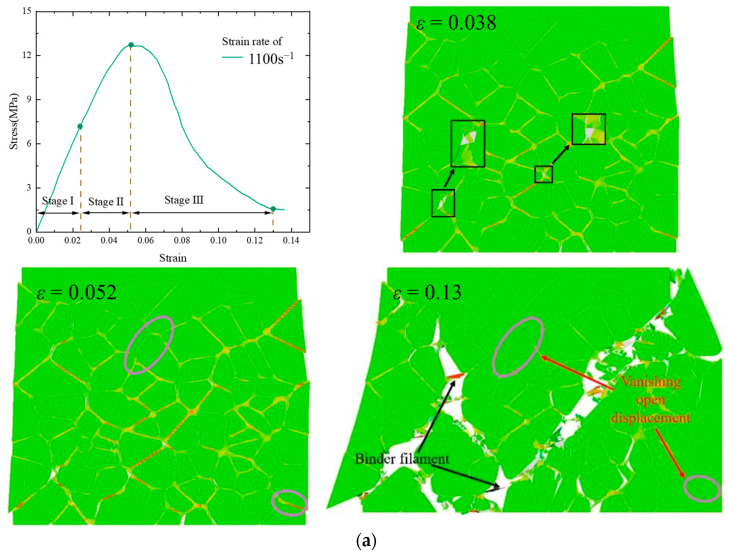

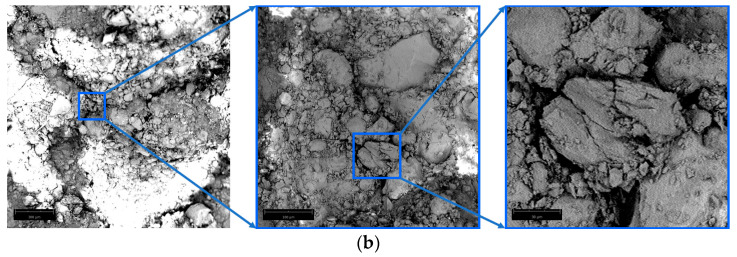
(**a**) Stress–strain curve and different strain clouds at a strain rate of 1100 s^−1^: (**b**) experimental observations.

The mechanical properties of the PBXs under high-strain-rate compressive loading exhibited non-linear variations and complex damage evolution. The strain and damage evolution diagrams were extracted from the numerical model subjected to a loading strain rate of 1100 s^−1^ (Figure 7a). Overall, the compression process has three distinct stages: the elastic stage (stage I), nonlinear growth stage (stage II), and softening stage (stage III). During stage I, significant deformation occurs in the polymer binder. In stage II, microcracks initiate and gradually propagate, leading to a non-linear enhancement in the mechanical properties. Finally, in stage III, microcrack coalescence and penetration occur rapidly, thereby hindering the mechanical properties.

The strain cloud at a loading strain rate of 1100 s^−1^ is depicted in Figure 7a. Additionally, Figure 7b presents the experimentally obtained micro-morphology of the PBXs under compressive damage at a strain rate of 1100 s^−1^. In stage I, the binder exhibits viscoelastic properties and can withstand larger deformations, thereby inhibiting crack formation. In stage II, binder debonding is initiated along with the formation of microcracks; however, most particles and binders still bear external loads, thus decreasing the load-bearing capacity of the model. In stage III, peak stress is attained and an increased number of microcracks are formed, thereby reducing load-bearing capacity as the main cracks converge and penetrate to form principal cracks under continuous external loading. Throughout this process, binder cracking dominates within the principal crack damage path and is accompanied by minimal particle cracking. A phenomenon known as “pulling” occurs due to significant polymer deformation, which aligns with the observations from the literature [45] as well as experimental findings (Figure 7b). Furthermore, the “vanishing displacement” condition can be observed (*ε* = 0.13), which indicates unloading of the model—a phenomenon described in [46].

### 4.2. Effect of Strain Rate on Mechanical Behavior and Damage

**Figure 8:**
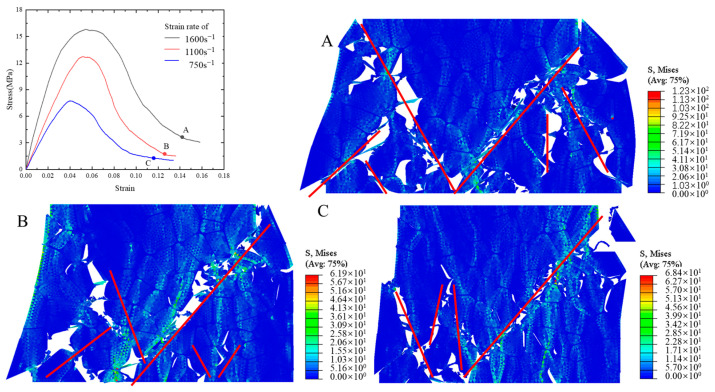
Damage path and von Mises stress distribution in the Y direction corresponding to points A, B, and C.

The stress–strain curves (Figure 8) demonstrate a pronounced strain rate effect at three different rates: 750 s^−1^, 1100 s^−1^, and 1600 s^−1^. This phenomenon has been documented in the existing literature [26,47]. The figure demonstrates a substantial increase in peak stress when the strain rate rises, whereas the enhancement in failure strain is not significant.

**Figure 9:**
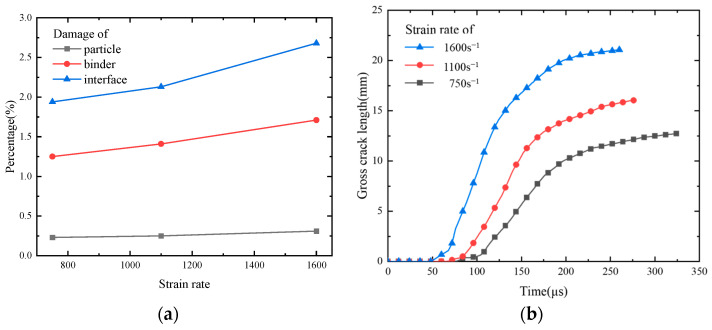
Damage at different strain rates: (**a**) percentage of damage caused to each component and (**b**) total length of visible cracks.

The failure paths of the PBXs observed at points A, B, and C for the three strain rates are shown in Figure 6 together with the cloud distribution of the von Mises equivalent stresses within the model. The failure paths, which are depicted as red lines in Figure 6, become increasingly intricate and inflict greater damage as the loading strain rate increases. The stress distribution cloud reveals that the stresses are predominantly concentrated in the vicinity of the failure path, while higher strain rates result in amplified internal stresses within the model. The damage ratio has been defined as the ratio of the number of failed cohesive elements to the total number of cohesive elements to obtain a more precise assessment of the damage level in the model. The damage ratios of the particles, binder, and interface are illustrated in Figure 9a, while Figure 9b depicts the variation in the overall crack length. The damage caused to the binder and interface exhibits a significant increase with an elevated strain rate (Figure 9a), while the damage ratio of the particles remains relatively unchanged. The dominant form of damage is interfacial debonding, which is accompanied by minor particle damage; this result aligns with the observed phenomenon in the experiments [43,48]. Figure 9b illustrates that the total length of the cracks increases with higher strain rates, indicating a corresponding increase in overall damage. This finding is consistent with previous literature reports [26].

### 4.3. Effect of Interface Strength on Mechanical Properties and Damage

**Figure 10:**
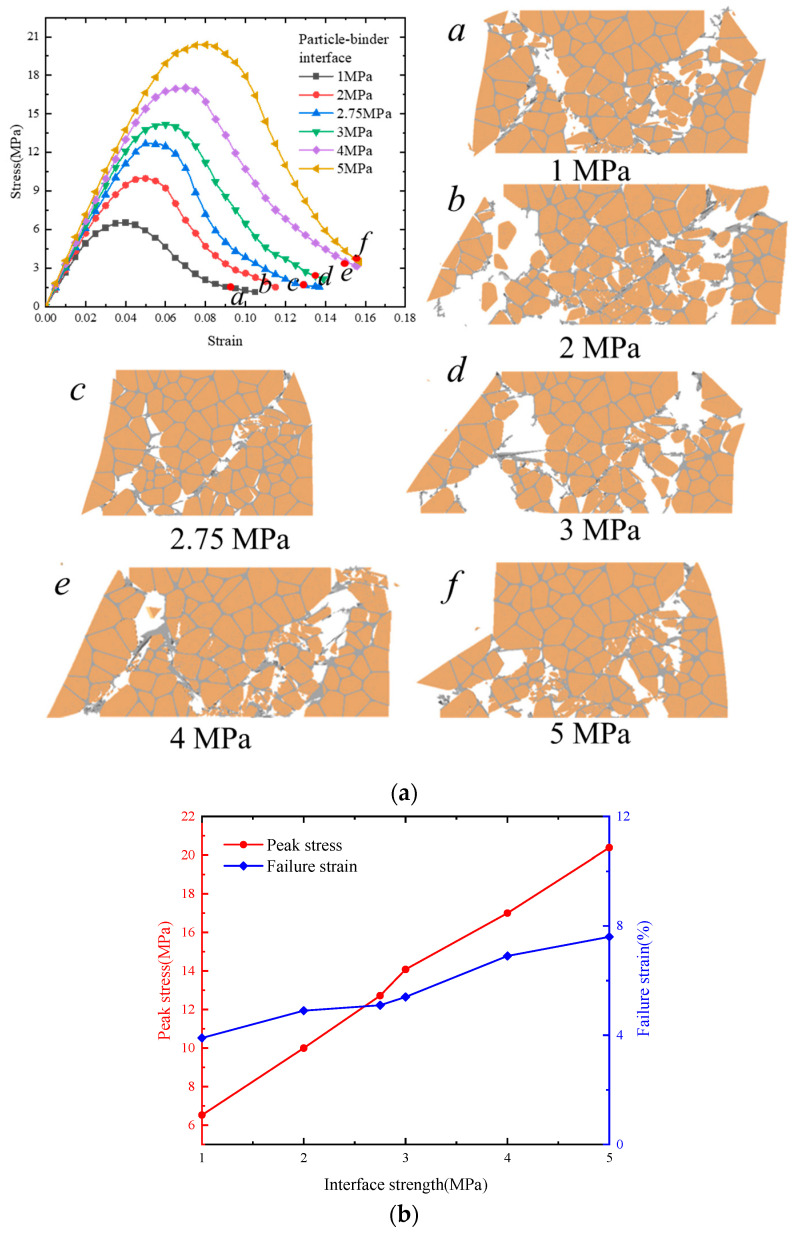
(**a**) Stress–strain curves at different particle–binder interface strengths; (**b**) peak stress and failure strain trends with interfacial strengths.

The interface between particles and binders plays a critical role in load transfer, thus directly influencing the mechanical properties of PBXs. Consequently, we conducted compression tests on a microscopic PBX model with different particle–binder interfacial strengths. The mechanical properties and damage modes of PBXs were investigated by varying the interfacial bond strength (1 MPa, 2 MPa, 3 MPa, 4 MPa, and 5 MPa), based on the previously established value of 2.75 MPa. Figure 10a shows the stress–strain curves of the PBXs with different interfacial bond strengths at a strain rate of 1100 s^−1^. The interfacial strength has a relatively smaller effect on the slope of the rising curve when compared to its effect on the peak stress. Meanwhile, the stress–strain curves of the peak stress and failure strain were plotted to gain a comprehensive understanding of how interfacial strength influences the mechanical properties of PBXs (Figure 10b). Both the peak stress and failure strain increase as interfacial strength increases, with a more pronounced effect being observed on the peak stress. This conclusion aligns with the findings reported in the relevant literature [38,39].

Interfacial bond strength has a significant effect on the damage patterns of PBXs, as demonstrated by Figure 10a. The damage pattern associated with the PBX microstructure encompasses interfacial debonding, binder tensile deformation, and particle damage, with interfacial debonding being the predominant factor. The damage degree of the particles gradually decreases as the interfacial bond strength increases to 1 MPa, 2 MPa, and 2.75 MPa. This phenomenon can be attributed to the premature interface failure caused by debonding when the particle–binder interface strength is low, which exerts external load on the particles and causes damage. When the interfacial bond strength is increased to 3 MPa, 4 MPa, and 5 MPa, the damage degree of the particles gradually escalates. This phenomenon can be attributed to the fact that as the interfacial bond strength approaches the internal bond strength of the particles (about 6 MPa), incremental particle damage occurs.

**Figure 11:**
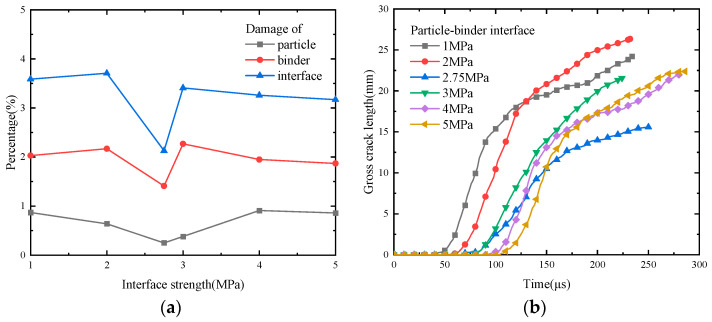
Damage modes of PBXs with different interface strengths: (**a**) percentage of damage caused to each component and (**b**) total length of visible cracks.

Figure 11 shows the statistical curves depicting the damage ratios and total lengths of the cracks at various locations of PBXs with different interfacial bond strengths. The damage ratio of each component is smaller when the interfacial strength is 2.75 MPa (Figure 11a). Additionally, the damage ratio of the particles tends to decrease and then increase, which aligns with the conclusion drawn in the previous section regarding the observed damage pattern. An increase in interfacial bond strength (Figure 11b) leads to a delayed damage initiation, thus indicating enhanced deformation resistance. At an interfacial bond strength of 2.75 MPa, the rate of damage accumulation decreases and the total crack length is smaller. These findings highlight that an appropriate interfacial bond strength not only confers excellent mechanical properties upon PBXs but also strengthens their damage resistance.

### 4.4. Effect of Particle Volume Fraction on Mechanical Properties and Damage

**Figure 12:**
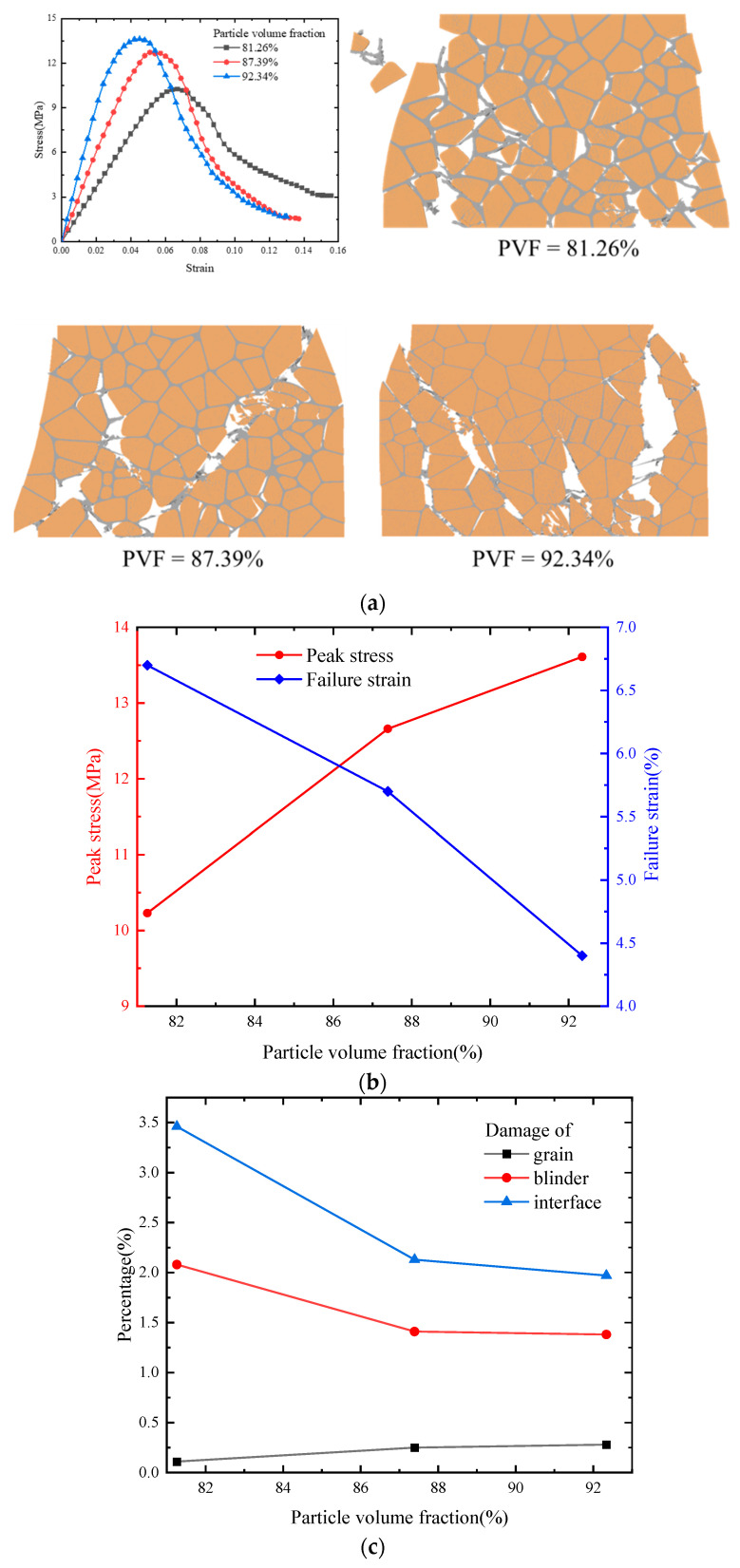

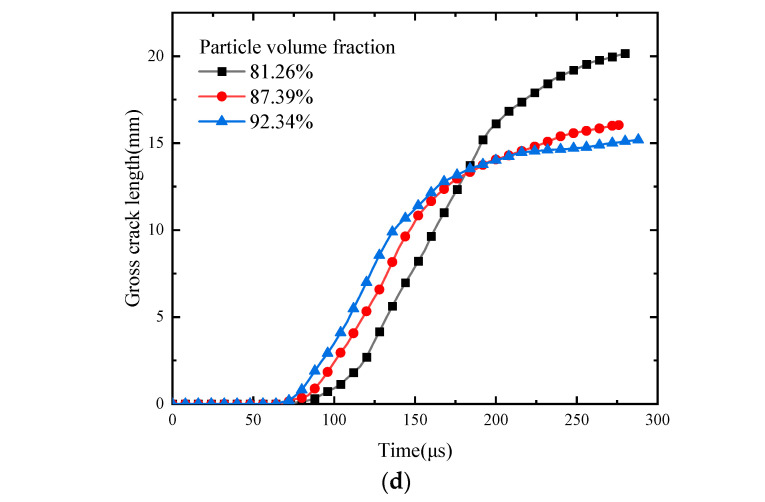
(**a**) Stress–strain curves for different particle volume fractions at 1100 s^−1^; (**b**) peak stress and failure strain curves; (**c**) percentage of damage caused to each component; (**d**) total length of visible cracks.

The particle volume fraction directly influences the dynamic mechanical properties of PBXs and the corresponding damage paths under external loading. Therefore, we established three microscopic models of PBXs with varying particle volume fractions (Figure 12). The stress–strain curves for the different particle volume fractions were obtained by subjecting them to compressive loading at a strain rate of 1100 s^−1^ (Figure 12a). The peak stress and failure strain curves are given in Figure 12b, which reveals that an increase in the particle volume fraction augments the slope of the stress–strain curve and thus enhances effective stiffness. Particle volume fraction exhibits a positive and negative correlation with peak stress and failure strain, respectively. Consequently, augmenting the particle fraction of a PBX can effectively enhance its load-carrying capacity, as reported in the existing literature [49,50].

Figure 12a shows the damage patterns of the PBXs with the different particle volume fractions under a strain rate of 1100 s^−1^. Although the final damage patterns and damage paths are different, they are still dominated by binder debonding and accompanied by minor particle damage. An increase in the binder volume fraction reduces the size of the damaged clump due to the viscoelastic nature exhibited by the binder. This property enables the PBX to endure larger deformations and a wider distribution of microcracks under continuous external loading.

The statistical curves associated with the damage ratio and the total length of the cracks at different locations of the PBXs with the various particle volume fractions are presented in Figure 12c,d. The damage ratio of the binder and interface decreases with an increase in particle volume fraction, while the damage ratio of the particles slightly increases (Figure 12c). The onset of and increase in the total crack length in Figure 12d exhibit a pronounced lag with decreasing particle volume fraction, resulting in a longer crack at the end. This behavior can be attributed to the higher binder fraction, increased strain resistance, inhibition of microcrack penetration, and presence of more uniformly distributed microcracks within the model.

## 5. Damage Evolution Equation Based on Microcracks

In the case of compression, it is assumed that damaged microcracks are uniformly distributed in all directions, and any interactions occurring between the microcracks and other micropores are disregarded. Therefore, the total length of the microcracks can be utilized to approximately determine the extent of damage caused to a PBX [51]:

(9) $$l_{c}=\sum_{i=1}^{n}l_{i}$$

where *n* is the total number of microcracks and *l_i_* is the length of the *i*th microcrack at time *t*. Figure 9 shows the corresponding curves depicting this relationship for various strain rates.

**Figure 13:**
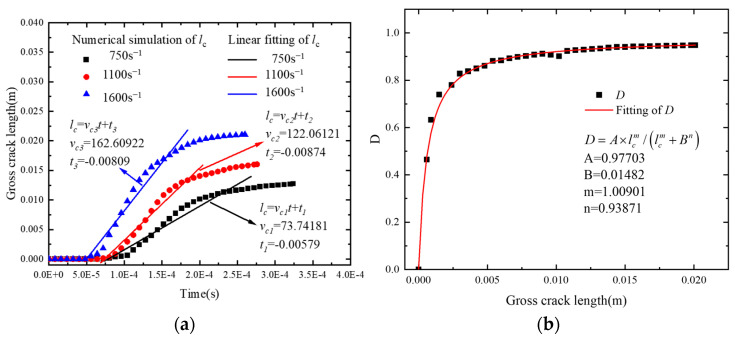
Law of microcrack propagation and corresponding macrodamage: (**a**) relationship between *l_c_* and t and (**b**) relationship between *D* and *l_c_*.

In Figure 13a, it is assumed that the microcracks propagate at a constant rate for different loading rates:

(10) $$l_{c}=v_{c}\langle t-t_{0}\rangle =v_{c}\langle \frac{\varepsilon }{\dot{\varepsilon }}-t_{0}\rangle$$

where ⟨ ⟩ represents the Macaulay brackets, *v_c_* is the equivalent crack propagation rate and is related to the loading rate [52], and *t*_0_ is the onset time of microcrack appearance. Based on the existing literature [51,53], the relationship between the macroscopic breakdown value, *D*, and the total length of microcracks, *l_c_*, was established:

(11) $$D=A\frac{l_{c}^{m}}{l_{c}^{m}+B^{n}}$$

where *A* is the damage coefficient; *B* is a constant related to the structure of the material, which can be interpreted as a structural parameter; and m and n are exponentials. Meanwhile, the *D*-*l*_c_ relationship was determined by fitting (Figure 13b).

By substituting Equation (10) into Equation (11), the relationship between the macroscopic damage value, *D*, and the macroscopic strain, *ε*, and strain rate, $\dot{\varepsilon }$, can be obtained via the following damage evolution equation:

(12) $$D=\frac{A{\langle \varepsilon -t_{0}\dot{\varepsilon }\rangle }^{m}}{{\langle \varepsilon -t_{0}\dot{\varepsilon }\rangle }^{m}+B^{n}{\left(\dot{\varepsilon }/v_{c}\right)}^{m}}$$

**Figure 14:**
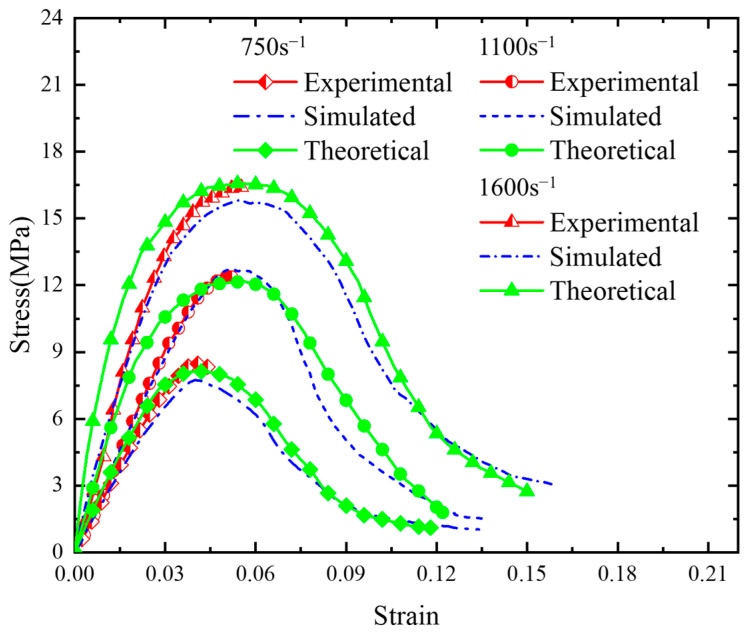
Comparison of experimental, simulated, and theoretical stress–strain curves.

To predict the macroscopic mechanical behavior of the PBX, we developed a damaged viscoelastic model:

(13) $$\sigma (t)=(1-D)\int_{0}^{t}E(t-\tau )\frac{\partial \varepsilon (\tau )}{\tau }d\tau$$

where E(t) is the modulus relaxation function. An *i*-order Prony series representation of the relaxation modulus can be written as follows:

(14) $$E(t)=\sum_{i=1}^{N}E_{i}\exp\left(-\frac{t}{\tau_{i}}\right)+E_{\infty }$$

where $E_{\infty }$ is the equilibrium modulus (taken as 1 MPa in this paper) while *E_i_* and *τ_i_* are the relaxation modulus and relaxation time, respectively. The relaxation modulus and relaxation time of the PBX obtained by fitting the experimental data are given in Table 3 [43], and the stress–strain curves obtained based on these parameters are in good agreement with the experimentally obtained and numerically simulated results (Figure 14).

## 6. Conclusions

This study systematically investigated the dynamic mechanical properties and damage behavior of PBXs with high particle volume fractions via FEM with a cohesive element embedded at the interface. Microstructural models revealed that the failure mechanism of a PBX under dynamic loading involves the initiation and gradual propagation of microcracks at the particle–matrix interface due to external loading, thereby leading to the formation of percolation cracks under continuous external loading.

The effects of strain rate, interfacial strength, and particle volume fraction on the mechanical properties and damage behavior of a PBX under dynamic loading were thoroughly investigated. Strain rate exerts a significant influence on both the mechanical properties and damage behavior of the material. Specifically, an increase in strain rate leads to a notable enhancement in the peak strength of the stress–strain curve, while failure strain remains relatively unchanged. Moreover, overall damage is significantly exacerbated with the increasing strain rate and primarily concentrated at the particle–matrix interface.

The effect of interface strength on the mechanical properties and damage resistance is substantial. As the interfacial strength increases from 1 MPa to 5 MPa, both peak strength and failure strain exhibit an upward trend; however, the overall damage level first decreases and then increases. This can be attributed to the premature interfacial damage that occurs between 1 MPa and 2.75 MPa, which leads to particle stress that can further increase due to the gradual rise in interfacial strength that occurs at a particle bond strength between 2.75 MPa and 5 MPa. Furthermore, the particle volume fraction has a significant effect on both the mechanical properties and damage level, which in turn are affected by the particle volume fraction. An increase in the volume fraction leads to an increase in the peak intensity but a decrease in the failure strain. The overall damage level tends to decrease, while the degree of particle damage increases.

The multi-scale damage evolution behavior was characterized, and a viscoelastic model incorporating damage was created by developing a correlation equation for the evolution of macroscopic and microscopic damages. This enhanced model provides a more accurate representation of the mechanical response of PBXs under dynamic loading. This paper also provides an effective numerical technique to study the mechanical properties and microscopic damage behavior of PBXs under dynamic loading.

## Figures and Tables

**Table 1:** Parameters associated with the Prony series of the polymer binder. Units for *E_i_* and *τ_i_* are MPa and s, respectively.

i	Log *τ_i_*	Log *E_i_*	Log *K_i_*	Log *G_i_*	i	Log *τ_i_*	Log *E_i_*	Log *K_i_*	Log *G_i_*
1	−9	1.708	2.231	1.246	9	−1	0.344	0.867	−0.118
2	−8	1.543	2.066	1.081	10	0	0.096	0.619	−0.366
3	−7	1.467	1.990	1.005	11	1	−0.534	−0.011	−0.996
4	−6	1.316	1.839	0.854	12	2	−0.285	0.238	−0.747
5	−5	1.134	1.657	0.672	13	3	−1.193	−0.670	−1.655
6	−4	0.996	1.519	0.534	14	4	−0.632	−0.109	−1.094
7	−3	0.775	1.298	0.313	15	5	−1.472	−0.949	−1.934
8	−2	0.616	1.139	0.157					

**Table 2:** Parameters associated with cohesive elements of material interfaces, where *K*, *σ*, and *G* represent cohesive stiffness, cohesive strength, and fracture energy, respectively.

Cohesive Element Type	*K* (MPa/mm)	*σ* (MPa)	*G* (N/mm)
Particle	1800	6	0.01
Binder	900	7.5	0.15
Particle–binder interface	800	2.75	0.012

**Table 3:** Relaxation Prony terms for a PBX.

Element	1	2	3	4	5
$\tau_{i}$ (s)	1 × 10^8^	1 × 10^7^	1 × 10^6^	1 × 10^5^	∞
$E_{i}$ (MPa)	297.17	221.82	175.39	78.34	1.66

## Data Availability

The original contributions presented in this study are included in the article. Further inquiries can be directed to the corresponding author.
